# Treatment of Acute Lymphoblastic Leukemia from Traditional Chinese Medicine

**DOI:** 10.1155/2014/601064

**Published:** 2014-05-22

**Authors:** Ya-Li Hsiao, Pei-Chun Chang, Hung-Jin Huang, Chia-Chen Kuo, Calvin Yu-Chian Chen

**Affiliations:** ^1^Department of Biomedical Informatics, Asia University, Taichung 41354, Taiwan; ^2^School of Medicine, College of Medicine, China Medical University, Taichung 40402, Taiwan

## Abstract

Acute lymphoblastic leukemia (ALL) is a cancer that immature white blood cells continuously overproduce in the bone marrow. These cells crowd out normal cells in the bone marrow bringing damage and death. Methotrexate (MTX) is a drug used in the treatment of various cancer and autoimmune diseases. In particular, for the treatment of childhood acute lymphoblastic leukemia, it had significant effect. MTX competitively inhibits dihydrofolate reductase (DHFR), an enzyme that participates in the tetrahydrofolate synthesis so as to inhibit purine synthesis. In addition, its downstream metabolite methotrexate polyglutamates (MTX-PGs) inhibit the thymidylate synthase (TS). Therefore, MTX can inhibit the synthesis of DNA. However, MTX has cytotoxicity and neurotoxin may cause multiple organ injury and is potentially lethal. Thus, the lower toxicity drugs are necessary to be developed. Recently, diseases treatments with Traditional Chinese Medicine (TCM) as complements are getting more and more attention. In this study, we attempted to discover the compounds with drug-like potential for ALL treatment from the components in TCM. We applied virtual screen and QSAR models based on structure-based and ligand-based studies to identify the potential TCM component compounds. Our results show that the TCM compounds adenosine triphosphate, manninotriose, raffinose, and stachyose could have potential to improve the side effects of MTX for ALL treatment.

## 1. Introduction

Dihydrofolate reductase (DHFR) is essential in cellular metabolism and cell growth. It catalyzes the conversion of dihydrofolate into tetrahydrofolate which is a carrier for the methyl group. The methyl group carried by tetrahydrofolate is required for de novo synthesis of varieties of essential metabolites including amino acids, lipids, pyrimidines, and purines. Methotrexate (MTX), a folate antagonist, arrests cell growth by competitively binding to DHFR, thereby, blocking de novo synthesis of nucleotide precursors and inhibiting DNA synthesis [1]. MTX has been found to be useful as an antineoplastic and immunosuppressive agent because it inhibits the proliferation of rapidly dividing malignant [2].

MTX tightly binding on DHFR is one of the most widely used drugs in cancer treatment and is especially effective in the treatment of acute lymphocytic leukemia [3]. In addition, its folate analogue is widely used in the treatment of acute lymphoblastic leukemia (ALL) [4], ovarian cancer [5], osteosarcoma [6], rheumatoid arthritis [7], psoriasis [8], and inflammatory bowel disease [9] and for prevention of graft-versus-host disease after transplantation [10].

In the cells, MTX acts by inhibiting two enzymes. First, as an analog of folate, MTX is a powerful competitive inhibitor with 1000-fold more potent than the natural substrate of DHFR. DHFR is responsible for converting dihydrofolate (FH2) to their active form tetrahydrofolate (FH4), which is a substrate of thymidylate synthase (TS). Second, MTX is converted to active methotrexate polyglutamates (MTX-PGs) by folylpolyglutamate synthase [11, 12]. The polyglutamated forms of MTX inhibit TS directly. Due to these inhibitions, the cells will not be capable of de novo synthesis of purines and thymidylate, and thus DNA synthesis will be inhibited [13].

**Figure 1:**
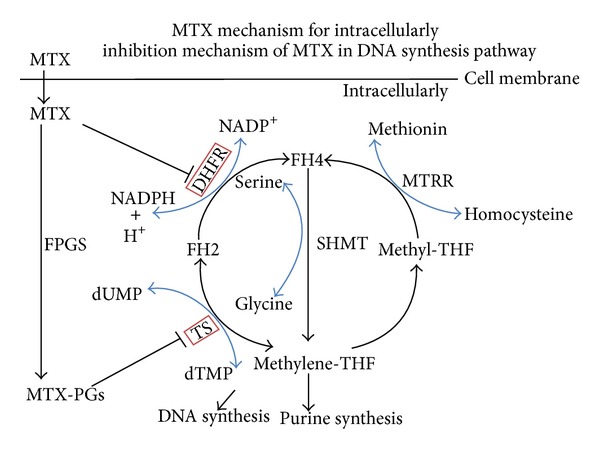
Inhibition mechanism of MTX in DNA synthesis pathway. MTX: methotrexate; FPGS: folylpolyglutamate synthetase; MTX-PGs: methotrexate polyglutamates; DHFR: dihydrofolate reductase; TS: thymidylate synthase; FH4: tetrahydrofolate; FH2: dihydrofolate; Methylene-THF: 5,10-methylenetetrahydrofolate; Methyl-THF: 5-methyltetrahydrofolate; dUMP: deoxyurindine-5′-monophosphate; dTMP: deoxythymidine-5′-monophosphate; MTRR: methionine synthase reductase; SHMT: serine hydroxymethyltransferase.

The primary action of MTX is inhibition of the enzyme DHFR, which converts dihydrofolate (FH2) to tetrahydrofolate (FH4) [11, 14]. MTX-PGs exert a stronger inhibition of DHFR and TS [15–17]. Thus, through direct inhibition by MTX and due to lack of FH4 and accumulation of FH2, deoxythymidine monophosphate synthesis and purine de novo synthesis is blocked, which eventually lead to leukemic cell death, bone marrow suppression, gastrointestinal mucositis, liver toxicity, and, rarely, alopecia [14, 15, 18, 19]. In fact, both MTX and natural folates undergo polyglutamylation catalyzed by the enzyme folylpolyglutamyl synthase. The MTX-PGs ensure intracellular retention and, furthermore, increase the affinity for the MTX-sensitive enzymes [16, 18, 20] (Figure 1).

However, MTX may lead to acute renal cytotoxicity [21] which is serious and potentially fatal in the spinal canal and may occur after the administration of neurotoxicity [22–25] and hematological toxicity [26] caused by animal somatic cells and human bone marrow chromosomal lesions [27] which led to the hematopoietic system abnormalities [28], gastrointestinal toxicity [29] made multiorgan dysfunction [30], nephrotoxicity [31] made renal failure [31, 32], and hepatotoxicity made liver fibrosis [33]. Higher concentrations of long-chain MTX-PGs have been in the risk of gastrointestinal and hepatic toxicity [12, 34, 35]. Thus, the lower toxicity drugs are necessary to be developed. Recently, the increasing numbers of mechanisms of different diseases have been clarified to detect the helpful target protein for diseases treatment [36–49], and diseases treatments with traditional Chinese medicine (TCM) as complements are getting more and more attention. The compounds extracted from traditional Chinese medicine have displayed their potential as lead compounds against tumors [50–54], stroke [55–58], viral infection [59–63], metabolic syndrome [64–66], diabetes [67], inflammation [62], and other diseases [68, 69]. For this trend, we attempted to discover the compounds with drug-like potential and lower toxicity for ALL treatment from the components in traditional Chinese medicine.

## 2. Materials and Methods

### 2.1. Virtual Screening

The receptors, human dihydrofolate reductase (DHFR) and human thymidylate synthase (TS) proteins were downloaded from Protein Data Bank of 1U72 (PDB ID: 1U72) [70] and 1HVY (PDB ID: 1HVY) [71]. We adopted the traditional Chinese medicine formulas that treat acute lymphoblastic leukemia from database “Shanghai Innovative Research Center of Traditional Chinese Medicine” (http://www.sirc-tcm.sh.cn/en/index.html) [72]. The component compounds of these formulas were integrated with the herbs data from the TCM Database@Taiwan [73] and became the ALL disease-specific compound library. Virtual screening of candidates from the compound library was conducted using the LigandFit Module of DS 2.5 under the Chemistry at HARvard Macromolecular Mechanics (CHARMm) force field. DockScore was selected as output values. Candidates were ranked according to DockScore and pharmacokinetic characteristics including absorption, solubility, blood brain barrier (BBB), and plasma protein binding (PPB) were predicted by ADMET protocols for each candidate.

### 2.2. 2D-Quantitative Structure Activity Relationship (2D-QSAR) Models

**Figure 2:**
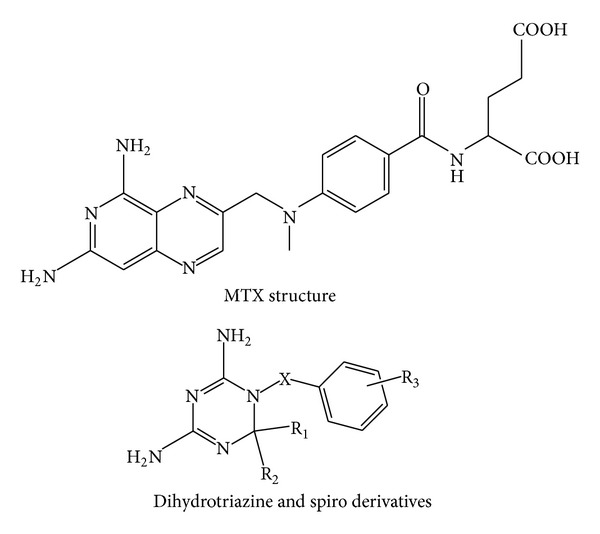
Chemical structure of DHFR inhibitors [40].

In this study, 45 candidates (Figure 2) with known experimental pIC50 values [74] that have inhibitory activities toward DHFR were used in the QSAR studies (Table 1). The 45 known inhibitors were randomly divided into a training set of 36 candidates and a test set of 9 candidates. The chemical structures of these candidates were drawn by ChemDraw Ultra 10.0 (CambridgeSoft Inc., USA) and transformed to 3D molecule models by Chem3D Ultra 10.0 (CambridgeSoft Inc., USA). Molecular descriptors for each candidate were calculated using the DS 2.5 Calculate Molecular Property Module. Genetic function approximation (GFA) model was used to select representative descriptors that correlated (*r* ^2^ > 0.8) to bioactivity (pIC50) which were used to construct 2D-QSAR models. The training set was used to construct multiple linear regression (MLR), support vector machine (SVM), and Bayesian network (BN) models. The test set was used to test the accuracy of these models.

#### 2.2.1. Multiple Linear Regression (MLR) Model

Multiple linear regression [75] attempts to model the relationship between two or more explanatory variables and a response variable by fitting a linear equation to observed data. The model was built in the form of equation as follows:

(1) $$\begin{array}{c} \text{pI}{\text{C}}_{50}=a_{0}+a_{1}x_{1}+a_{2}x_{2}+\cdots +a_{n}x_{n}, \end{array}$$

where *x*_*i*_ represents the *i*th molecular descriptor and *a*_*i*_ is its fitting coefficient. The generated MLR model was validated with test dataset. The square correlation coefficients (*R* ^2^) between predicted and actual pIC50 of the training set was used to verify accuracy of the model. This building model was applied to predict the pIC50 values of the TCM candidates.

#### 2.2.2. Support Vector Machine (SVM) Model

SVM implement classification or regression analysis with linear or nonlinear algorithms [76]. The algorithm identifies a maximum-margin hyper-plane to discriminate two class training samples. Samples on the margin are called the support vectors. Lagrange multipliers and kernels were introduced to form the final pattern separating regression model. In this study, LibSVM [77–79] package was selected to build our regression SVM model. The selected kernel was the Gaussian radial basis function kernel equation:

(2) $$\begin{array}{c} K\left(x_{i},x_{k}\right)=\exp\left[\frac{{||x_{i}-x_{k}||}^{2}}{2\sigma^{2}}\right]. \end{array}$$

Cross-validation of the SVM model was also conducted following the default settings in LibSVM [80]. The generated regression SVM model was validated with test dataset. The square correlation coefficients (*R* ^2^) between predicted and actual pIC50 of the training set was used to verify accuracy of the model. This building model was applied to predict the pIC50 values of the TCM candidates.

#### 2.2.3. Bayesian Network Model

We used the Bayes Net Toolbox (BNT) in Matlab (https://code.google.com/p/bnt) to create Bayesian network model [81] by the training data set. After data discretization, we applied linear regression analysis for each pIC50 category in the training dataset. For the *i*th pIC50 category with *n* candidates, let *y*_*ij*_ and *x*_*ijp*_ represent the pIC50 value and the *p*th descriptor value in the *j*th ligand, respectively. The regression model of the data sets {*y*_*ij*_, *x*_*ij*1_,…, *x*_*ijp*_}_*j*=1_^*n*^ is formulated as

(3) $$\begin{array}{c} y_{i}=X_{i}\beta_{i}+\varepsilon_{i}, \end{array}$$

where

(4) $$\begin{array}{c} y_{i}=\left[\begin{array}{c} y_{i1}\\ y_{i2}\\ \vdots \\ y_{in} \end{array}\right],\;\;X_{i}=\left[\begin{array}{ccc} x_{i11} & \cdots & x_{i1p}\\ x_{i21} & \cdots & x_{i2p}\\ \vdots & \vdots & \vdots \\ x_{in1} & \cdots & x_{inp} \end{array}\right], \end{array}$$

and *β*_*i*_ and *ε*_*i*_ are the regression coefficients and error term in the *i*th pIC50 category. We used ordinary least squares to estimate the unknown regression coefficient *β*_*i*_:

(5) $$\begin{array}{c} {\hat{\beta }}_{i}={\left(X_{i}^{T}X_{i}\right)}^{-1}X_{i}^{T}y_{i}. \end{array}$$

The Banjo (Bayesian network inference with Java objects) is software for structure learning of static Bayesian networks (BN) [82]. It is implemented in Java. We used training dataset to discover the relationships in the BN structure among the descriptors and the pIC50 by the Banjo package. After that, we used test data to assess the accuracy of our algorithm. For the test data *D*, the pIC50 category (*k*) is predicted by the following formula:

(6) $$\begin{array}{c} k=\underset{i=1}{\overset{n}{\arg\;\max\;}}P\left(i\;|\;D\right), \end{array}$$

where *i* represented the *i*th category of pIC50 and *n* represented the total number of the pIC50 categories. The marginal probability *P*(*i*∣*D*) can be calculated by BNT module. Finally, the pIC50 value is calculated as follows:

(7) $$\begin{array}{c} \text{pIC}50=X_{k}{\hat{\beta }}_{k}. \end{array}$$

The square correlation coefficients (*R* ^2^) between predicted and actual pIC50 of the training set were used to verify accuracy of the model. This building model was applied to predict the pIC50 values of the TCM candidates.

### 2.3. 3D-Quantitative Structure Activity Relationship (3D-QSAR) Models

Comparative molecular field analysis (CoMFA) and comparative molecular similarity indices analysis (CoMSIA) were performed by Sybyl-X 1.1.1 (Tripos Inc., St. Louis, MO, USA) for DHFR inhibitors. Lennard-Jones potential and Coulomb potential were employed to calculate steric and electrostatic interaction energies. The two 3D-QSAR models were further evaluated by cross-validated correlation coefficient (*q* ^2^) and non-cross-validated correlation coefficient (*r* ^2^). The correlation between the force field and biological activities was calculated by partial least squares (PLSs) method.

**Figure 3:**
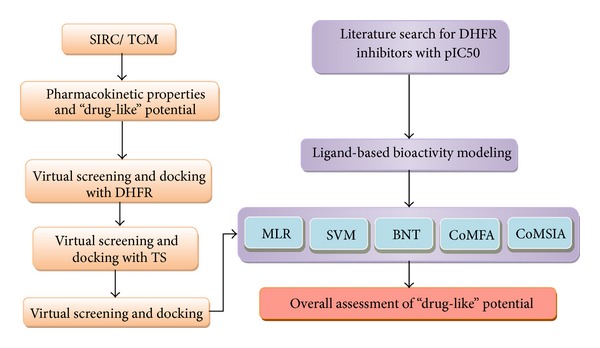
The experimental flowchart.

The flowchart for the entire experimental procedure for TCM candidates screening is illustrated in Figure 3.

## 3. Results and Discussion

### 3.1. Virtual Screening

**Figure 4:**
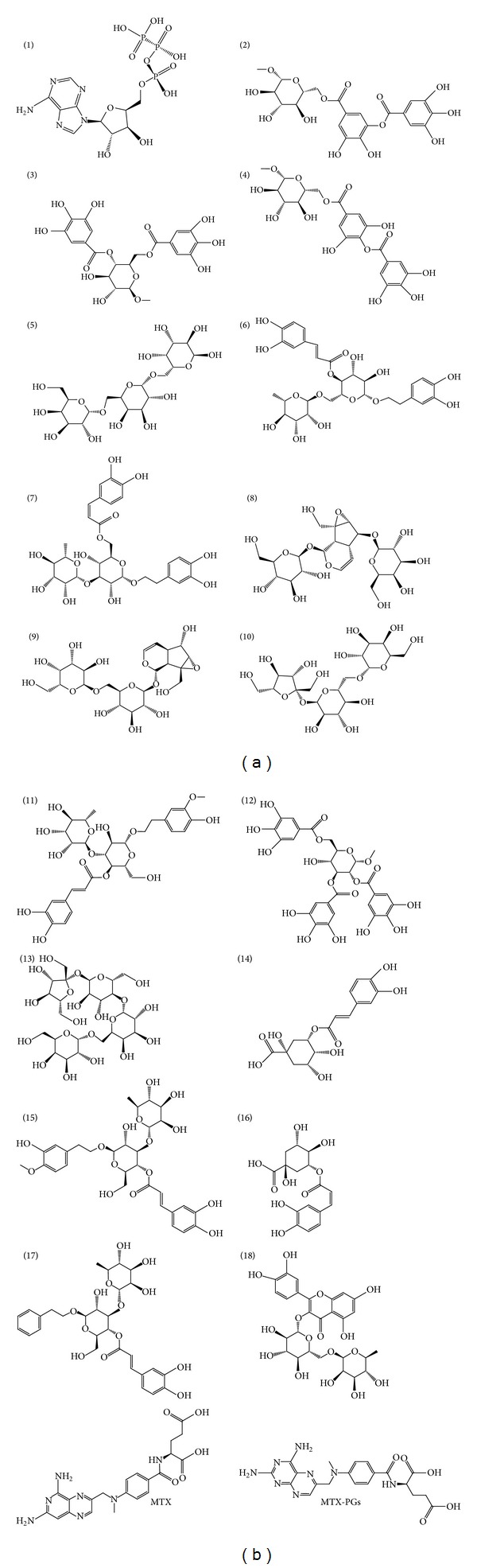
(a)-(b) The chemical scaffolds of MTX, MTX-PGs, and TCM candidates for acute lymphoblastic leukemia treatment.

The virtual screening was performed by the LigandFit Module of DS 2.5 in force field of CHARMm. The receptor binding sites were defined by the binding position of MTX on DHFR protein and by the binding position of MTX-PGs on TS protein. The compounds from our library were docked into the two receptors. In this protocol, the receptors were fixed, and the ligands that complement the binding sites were flexible in energy minimization process. The control compound used in this study was MTX which contains aromatic and heterocyclic rings (Figure 4).

The top eighteen results from DHFR docking score are tabulated in Table 2. The TS docking score for the eighteen candidates are also tabulated in Table 2. All the eighteen TCM candidates had higher Dock Scores than the control methotrexate (MTX) and MTX-PGs. Chemical scaffolds of MTX, MTX-PGs, and the eighteen TCM candidates are shown in Figure 4. Adsorption, solubility, hepatotoxicity, and plasma protein binding were assessed to evaluate pharmacokinetic properties of the selected candidates (Table 3). Considering the factor of hepatotoxicity, we selected the TCM compounds adenosine triphosphate, manninotriose, raffinose, and stachyose for advanced study. MTX and TCM candidates had very poor absorption for human intestine. Binding strength of the ligands to carrier proteins in the blood stream is indicated by the plasma protein binding (PPB) value [21]. MTX has more than 90% for PPB but adenosine triphosphate, manninotriose, raffinose, and stachyose were less than 90% for PPB.

**Figure 5:**
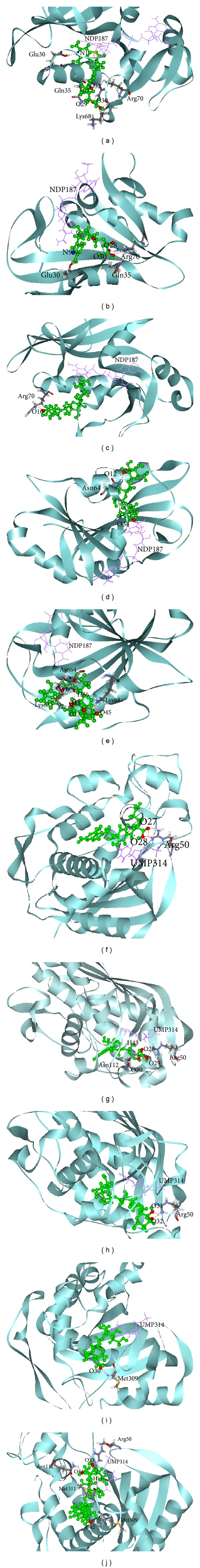
Docking pose of MTX and TCM candidates with DHFR for (a), (b), (c), (d), and (e). Docking pose of MTX-PGs with TS for (f), (g), (h), (i), and (j). TCM candidates are shown in cyan. The cofactors are shown in purple. In H-bond interactions, nitrogen atoms are shown in blue, hydrogen atoms are shown in gray, oxygen atoms are shown in magenta, hydrogen bonds are shown in red dotted line, pi bonds are shown in orange solid line. (a) MTX, (b) and (g) adenosine triphosphate, (c) and (h) manninotriose, (d) and (i) raffinose, (e) and (j) stachyose, and (f) MTX-PGs.

**Figure 6:**
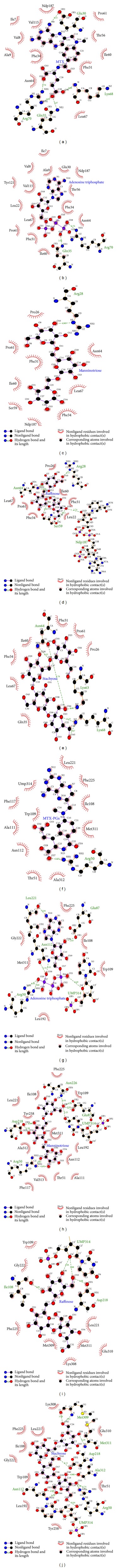
The Ligplot analysis of hydrophobic interactions between DHFR and TCM candidates and between TS and TCM candidates. (a) MTX with DHFR, (b) and (g) adenosine triphosphate with DHFR and TS, (c) and (h) manninotriose with DHFR and TS, (d) and (i) raffinose with DHFR and TS, (e) and (j) stachyose with DHFR and TS, and (f) MTX-PGs with DHFR and TS. Bonds: ligand bonds, nonligand bonds, hydrogen bonds, and hydrophobic are shown in purple, orange, olive green, and brick red, respectively. Atoms: nitrogen, oxygen, carbon, and sulfur are shown in blue, red, black, and yellow, respectively.

Ligand-receptor interactions during docking are shown in Figures 5 and 6. MTX docked on DHFR (Figure 5(a)) through four hydrogen bondings of Glu30, Gln35, Lys68, and Arg70. Adenosne triphosphate formed three H-bonds with Glu30, Gln35, and Arg70 (Figure 5(b)). Manninotriose formed H-bond with Arg28 (Figure 5(c)). Raffinose formed H-bonds with Asn64 and NDP (Figure 5(d)). Stachyose formed H-bonds with Lys63, Asn64, and Lys68 (Figure 5(e)). MTX-PGs docked on TS (Figure 5(f)) by single H-bond with Arg50. Adenosne triphosphate, manninotriose, and stachyose docked on TS (Figures 5(g), 5(h), and 5(j)) by single H-bond with Arg50. Raffinose docked on TS by single H-bond with Met309 (Figure 5(i)).

Analysis of hydrophobic interactions showed that MTX docking on DHFR was more stable than the TCM candidates. Comparing with chemical structures of the TCM candidates, it could be attributed to the larger size for MTX docking on DHFR (Figures 6(a), 6(b), 6(c), 6(d), and 6(e)). However, the TCM candidates docking on TS were more stable than MTX-PGs due to hydrophobic interactions (Figures 6(f), 6(g), 6(h), 6(i), and 6(j)).

### 3.2. Bioactivity Prediction Using QSAR Models

QSAR models were constructed using known DHFR inhibitors [40] and applied for predicting molecular properties of the TCM ligands. Molecular descriptors associated with bioactivity including BD_Count, Num_RotatableBonds, CHI_V_1, IAC_Mean, JX, JY, SC_3_C, Jurs_FNSA_1, Jurs_RPCS, Jurs_SASA, and Shadow_Xlength were used to construct MLR model, SVM model, and Bayesian network model.

Our MLR model was as follows.

GFATempModel_1 = 31.623 + 2.5173  ∗  HBD_Count − 0.47471  ∗  Num_RotatableBonds − 1.7664  ∗  CHI_V_1 − 12.997  ∗  IAC_Mean − 45.669  ∗  JX + 36.62  ∗  JY + 0.11612  ∗  SC_3_C + 18.941  ∗  Jurs_FNSA_1 − 4.8012  ∗  Jurs_RPCS + 0.029451  ∗  Jurs_SASA − 0.084377  ∗  Shadow_Xlength.

In CoMFA model, the steric fields were the primary contributing factor. In CoMSIA, various factors were considered and modeled. The optimum CoMSIA models were “EHA model” and “EHDA model” based on high *q* ^2^, high *r* ^2^, and low SEE values (Table 4). The “EHA model” was consisting of electrostatic field and hydrophobic and hydrogen bond acceptor. The “EHDA model” was consisting of electrostatic field and hydrophobic and hydrogen bond donor, and hydrogen bond acceptor. The CoMFA model and CoMSIA model of EHDA and of EHA were with ONC of 7, 11, and 12, respectively.

Experimental and predicted pIC50 values of 45 DHFR inhibitors using CoMFA and CoMSIA models are shown in Table 5. Residuals calculated from the differences between observed and predicted pIC50 values ranged between −0.3655 and 0.4311 for the CoMFA, between −0.411 and 0.589 for the CoMSIA with “EHA model,” and between −0.431 and 0.569 for CoMSIA with “EHDA model.”

**Figure 7:**
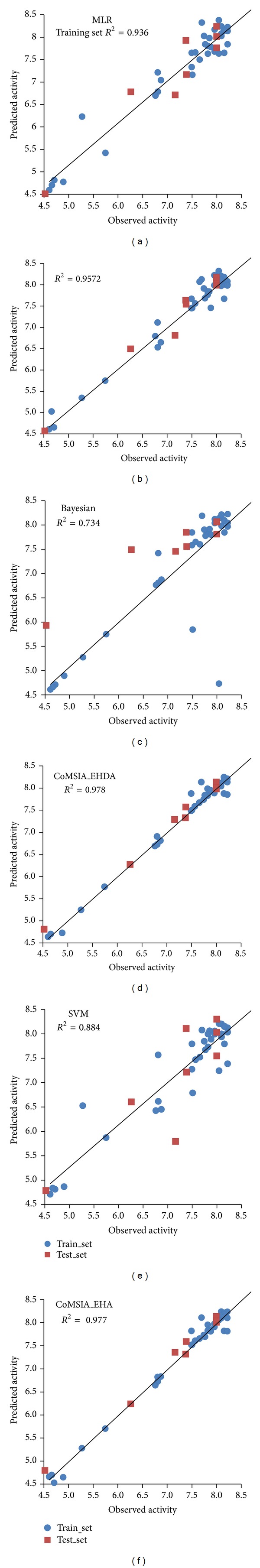
Correlation of observed and predicted activity (pIC50) using 2D-QSAR models and 3D-QSAR models. MLR, Bayesian network, and SVM were 2D-QSAR model. CoMFA, CoMSIA_EHDA, and CoMSIA_EHA were 3D-QSAR model.

The correlations between the predicted and actual bioactivity for DHFR inhibitors are shown in Figure 7. The *R* ^2^ values are 0.936 for MLR, 0.734 for Bayesian network, 0.884 for SVM, 0.957 for CoMFA, 0.977 for CoMSIA with EHA model, and 0.978 for CoMSIA with EHDA model implicate high correlation. High correlation coefficients validated the reliability of the constructed CoMFA and CoMSIA models. The predicted bioactivity values of TCM candidates by 2D-QSAR and 3D-QSAR models are listed in Table 6.

### 3.3. The Contour Maps of CoMFA and CoMSIA Models

**Figure 8:**
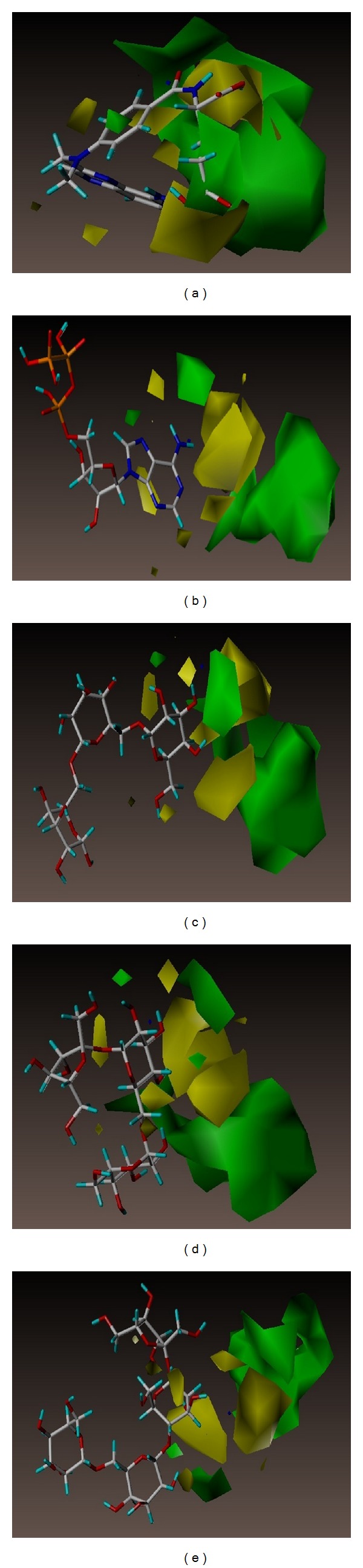
The CoMFA contour maps for DHFR. (a) MTX, (b) adenosine triphosphate, (c) manninotriose, (d) raffinose, and (e) stachyose. Green and yellow contours denote regions favoring and disfavoring steric fields, respectively. Blue and red contours denote regions favoring and disfavoring electrostatic fields, respectively.

**Figure 9:**
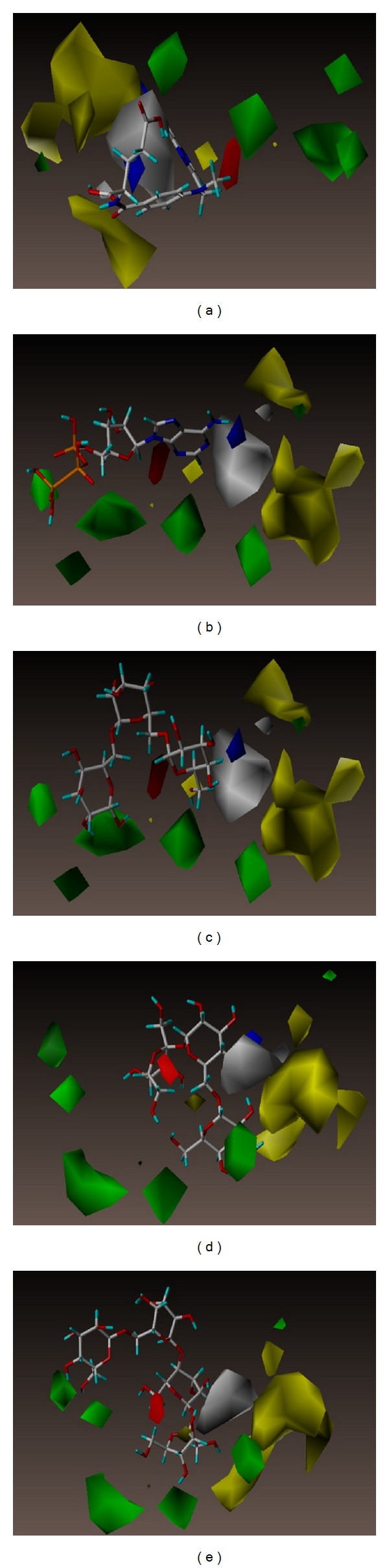
The CoMSIA contour maps of EHA model for DHFR. (a) MTX, (b) adenosine triphosphate, (c) manninotriose, (d) raffinose, and (e) stachyose. Blue and orange contours denote regions favoring and disfavoring electrostatic fields, respectively. Yellow and white contours denote regions favoring and disfavoring hydrophobic fields, respectively. Green and red contours denote regions favoring and disfavoring H-bond acceptor fields, respectively.

**Figure 10:**
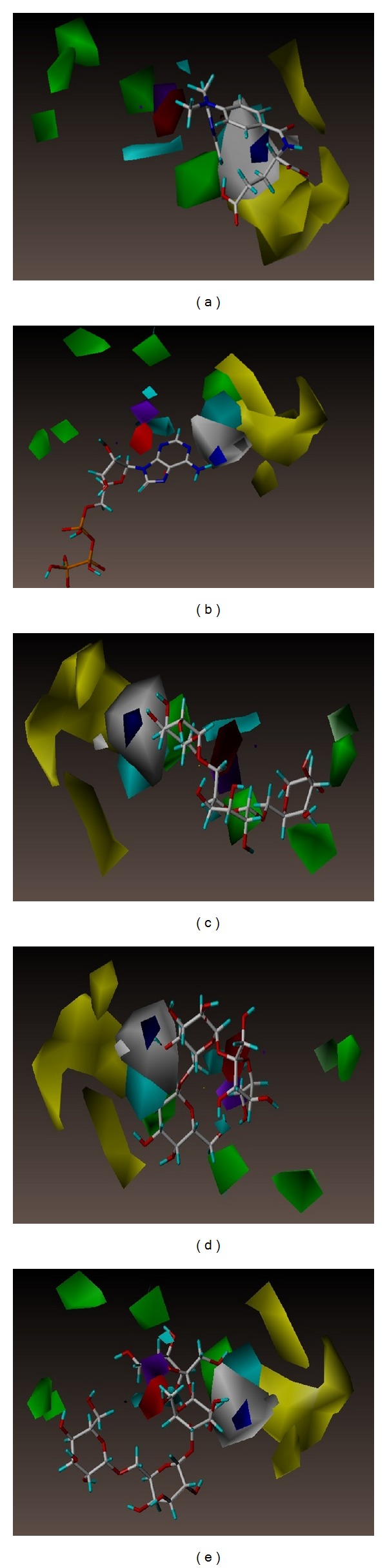
The CoMSIA contour maps of EHDA model for DHFR. (a) MTX, (b) adenosine triphosphate, (c) manninotriose, (d) raffinose, and (e) stachyose. Blue and orange contours denote regions favoring and disfavoring electrostatic fields, respectively. Yellow and white contours denote regions favoring and disfavoring hydrophobic fields, respectively. Green and red contours denote regions favoring and disfavoring H-bond acceptor fields, respectively. Cyan and purple contours denote regions favoring and disfavoring H-bond donor fields, respectively.

Ligand activities of MTX and the TCM candidates can be predicted based on the 3D-QSAR contour map, including features in steric field, hydrophobic field, and H-bond donor/acceptor characteristics. MTX and the TCM candidates contoured well to the steric features of the CoMFA in Figure 8. CoMSIA map provides more information with regard to bioactivity differences for “EHA model” and “EHDA model” in Figures 9 and 10, respectively. From the consistent results observed among the 3D-QSAR models validations, we inferred that adenosine triphosphate, manninotriose, raffinose, and stachyose of TCM candidates might have good biological activity for DHFR.

Contour to steric favoring and hydrophobic favoring regions was observed for adenosine triphosphate, manninotriose, raffinose, and stachyose. Consistent with the docking pose contour (Figures 8, 9, and 10), we propose that the four TCM candidates may maintain bioactivity for DHFR under dynamic conditions in physiological environments.

## 4. Conclusion

DHFR and TS proteins are key regulators in de novo synthesis of purines and thymidylate. Inhibiton of these proteins has the potential for treating acute lymphoblastic leukemia. In this study, we applied virtual screen and QSAR models based on structure-based and ligand-based methods in order to identify the potential TCM compounds. The TCM compounds adenosine triphosphate, manninotriose, raffinose, and stachyose could bind on DHFR and TS specifically and had low hepatotoxicity. These TCM compounds had potential to improve the side effects of MTX for ALL treatment.

## Figures and Tables

**Table 1:** Experimental pIC50 values for DHFR inhibitors [40].

Name	R1	R2	X	R3	pIC50
1	CH_3_	CH_3_	CH_2_	H	4.71
2	CH_3_	CH_3_	CH_2_	4′-CH_3_	4.6091
3∗	CH_3_	CH_3_	CH_2_	4′-OCH_3_	4.2306
4	CH_3_	CH_3_	CH_2_	4′-F	4.6615
5∗	CH_3_	CH_3_	CH_2_	4′-Cl	4.5243
6	CH_3_	CH_3_	CH_2_	3′,4′-diCl	4.8928
7∗	CH_3_	CH_3_	–O-CH_2_–	H	7.1612
8	CH_3_	C_2_H_5_	–O-CH_2_–	H	6.8097
9∗	H	c-Pr	–O-CH_2_–	H	6.2612
10	–(CH_2_)_3_–		–O-CH_2_–	H	6.8729
11	– (CH_2_)_4_–		–O-CH_2_–	H	6.762
12	– (CH_2_)_5_–		–O-CH_2_–	H	5.7471
13	– (CH_2_)_6_–		–O-CH_2_–	H	5.2733
14	– (CH_2_)_4_–		–O-CH_2_CH_2_–	H	7.5086
15	– (CH_2_)_5_–		–O-CH_2_CH_2_–	H	8.0458
16	– (CH_2_)_4_–		–O-(CH_2_)_3_-O–	H	7.699
17	– (CH_2_)_5_–		–O-(CH_2_)_3_-O–	H	7.4949
18	CH_3_	CH_3_	–O-(CH_2_)_3_-O–	H	8.2218
19	CH_3_	CH_3_	–O-(CH_2_)_4_-O–	H	7.5686
20	CH_3_	C_2_H_5_	–O-(CH_2_)_3_-O–	H	8.0969
21	H	c-Pr	–O-(CH_2_)_3_-O–	H	8.1549
22	– (CH_2_)_4_–		–O-(CH_2_)_3_-O–	H	8.699
23∗	– (CH_2_)_4_–		–O-(CH_2_)_4_-O–	H	7.3768
24	– (CH_2_)_5_–		–O-(CH_2_)_3_-O–	H	8.1549
25	– (CH_2_)_5_–		–O-(CH_2_)_4_-O–	H	6.8069
26	–(CH_2_)_6_–		–O-(CH_2_)_3_-O–	H	7.9586
27	–(CH_2_)_5_–		–O-(CH_2_)_3_-O–	F	7.8239
28	–(CH_2_)_5_–		–O-(CH_2_)_3_-O–	Cl	7.8539
29	–(CH_2_)_5_–		–O-(CH_2_)_3_-O–	NO_2_	7.8239
30	–(CH_2_)_5_–		–O-(CH_2_)_3_-O–	Me	7.7447
31	–(CH_2_)_5_–		–O-(CH_2_)_3_-O–	t-Bu	7.6576
32	–(CH_2_)_5_–		–O-(CH_2_)_3_-O–	OMe	8.2218
33∗	–(CH_2_)_5_–		–O-(CH_2_)_3_-O–	CN	8
34	–(CH_2_)_5_–		–O-(CH_2_)_3_-O–	COCH_3_	7.8861
35	–(CH_2_)_5_–		–O-(CH_2_)_3_-O–	SO_2_NH_2_	8.2218
36∗	–(CH_2_)_4_–		–O-(CH_2_)_3_-O–	F	8
37	–(CH_2_)_4_–		–O-(CH_2_)_3_-O–	Cl	8.1549
38	–(CH_2_)_4_–		–O-(CH_2_)_3_-O–	NO_2_	8.0969
39∗	–(CH_2_)_4_–		–O-(CH_2_)_3_-O–	Me	8
40	–(CH_2_)_4_–		–O-(CH_2_)_3_-O–	t-Bu	7.7696
41	–(CH_2_)_4_–		–O-(CH_2_)_3_-O–	OMe	7.9586
42	–(CH_2_)_4_–		–O-(CH_2_)_3_-O–	CN	8.0969
43	–(CH_2_)_4_–		–O-(CH_2_)_3_-O–	COCH_3_	8.0458
44∗	–(CH_2_)_4_–		–O-(CH_2_)_3_-N(Me)–	H	7.3872
45	–(CH_2_)_4_–		–O-(CH_2_)_3_–	H	7.4949
MTX					8.5229

*test set.

**Table 2:** DHFR and TS docking score of TCM candidates.

Index	TCM candidate	DHFR docking score	TS docking score
1	Adenosine triphosphate	226.6790	186.2170
2	Methyl 6-O-digalloyl-beta-D-glucopyranoside (II)	162.6260	154.1730
3	Methyl 4,6-di-O-galloyl-beta-D-glucopyranoside	153.7500	148.2880
4	Methyl 6-O-digalloyl-beta-D-glucopyranoside	151.7650	158.0350
5	Manninotriose	129.7870	114.6030
6	Forsythiaside	129.6030	27.9940
7	Isoacteoside	124.5900	30.6190
8	Rehmannioside B	119.9930	79.2920
9	Rehmannioside A	116.4330	71.3970
10	Raffinose	115.4940	134.2120
11	Cistanoside C	112.4270	—
12	Methyl 3,3,6-tri-O-galloyl-beta-D-glucopyranoside	109.9470	20.7830
13	Stachyose	107.0940	8.5760
14	Chlorogenic acid	103.8080	—
15	Jionoside D	103.5050	39.3430
16	Isochlorogenic acid	102.9470	—
17	Jionoside C	102.3940	—
18	Rutin	101.1310	78.816
∗	MTX	97.0960	—
∗∗	MTX-PGs	—	69.671

*control.

**Methotrexate polyglutamate.

**Table 3:** Predicted pharmacokinetic properties of TCM candidates and MTX.

Index	TCM candidate	Pharmacokinetic properties			
		Absorption	Solubility	Hepatotoxicity	PPB
1	Adenosine triphosphate	3	2	1	0
2	Chlorogenic acid	3	4	1	0
3	Cistanoside C	3	2	1	2
4	Forsythiaside	3	2	1	0
5	Isoacteoside	3	2	1	0
6	Isochlorogenic acid	3	4	1	0
7	Jionoside C	3	3	1	2
8	Jionoside D	3	2	1	2
9	Manninotriose	3	3	0	0
10	Methyl 4,6-di-O-galloyl-beta-D-glucopyranoside	3	2	1	0
11	Methyl 6-O-digalloyl-beta-D-glucopyranoside	3	2	1	0
12	Methyl 6-O-digalloyl-beta-D-glucopyranoside (II)	3	2	1	0
13	Methyl 3,3,6-tri-O-galloyl-beta-D-glucopyranoside	3	0	1	0
14	Raffinose	3	3	0	0
15	Rehmannioside A	3	4	1	0
16	Rehmannioside B	3	4	1	0
17	Rutin	3	1	1	2
18	Stachyose	3	1	0	0
Control	MTX	3	3	1	1

^1^Absorption (Human intestinal absorption), there are four prediction levels: 0 (good absorption), 1 (moderate absorption), 2 (poor absorption), 3 (very poor absorption).

^2^Solubility, there are gour prediction levels: 0 (extremely low), 1 (very low, but possible), 2 (low), 3 (good), 4 (optimal), 5 (too soluble), 6 (warning).

^3^Hepatotoxicity, there are four prediction levels: 0 (nontoxic), 1 (toxic).

^4^PPB (Plasma protein binding), there are there prediction levels: 0 (binding is <90%), 1 (binding is >90%), 2 (binding >95%).

**Table 4:** Partial Least Square (PLS) analysis for CoMFA and CoMSIA models.

	Cross Validation		Non-cross Validtion			Fraction				
	ONC	*q* ^2^	*r* ^2^	SEE	*F*	S	E	H	D	A
CoMFA										
	7	0.5250	0.9630	0.2590	136.2760	0.7970	0.2030	—	—	—

CoMSIA										
S	36	0.6350	0.9890	0.3040	19.6900	1.0000	0.0000	0.0000	0.0000	0.0000
E	—	—	—	—	—	—	—	—	—	—
H	2	0.6130	0.7760	0.5940	72.7070	0.0000	0.0000	1.0000	0.0000	0.0000
D	7	0.4180	0.7160	0.7130	13.3480	0.0000	0.0000	0.0000	1.0000	0.0000
A	1	0.0810	0.1600	1.1380	8.1640	0.0000	0.0000	0.0000	0.0000	1.0000
SE	37	0.6050	0.9890	0.3250	16.7260	0.9980	0.0200	0.0000	0.0000	0.0000
SH	2	0.5970	0.7790	0.5910	73.9120	0.3880	0.0000	0.6120	0.0000	0.0000
SD	36	0.6670	0.9890	0.3020	19.9580	0.6350	0.0000	0.0000	0.3650	0.0000
SA	30	0.7020	0.9890	0.2340	39.7820	0.7480	0.0000	0.0000	0.0000	0.2520
EH	7	0.6270	0.9540	0.2860	110.4680	0.0000	0.0500	0.9500	0.0000	0.0000
ED	7	0.4130	0.7090	0.7210	12.9020	0.0000	0.0180	0.0000	0.9820	0.0000
EA	2	0.0760	0.1830	1.1350	4.6860	0.0000	0.2000	0.0000	0.0000	0.8000
HD	2	0.5780	0.7940	0.5690	81.1680	0.0000	0.0000	0.7220	0.2780	0.0000
HA	2	0.5890	0.7910	0.5740	79.5410	0.0000	0.0000	0.7450	0.0000	0.2550
DA	9	0.4300	0.7290	0.7160	10.4430	0.0000	0.0000	0.0000	0.7800	0.2200
SHE	8	0.5850	0.9690	0.2400	139.5820	0.3570	0.0440	0.6000	0.0000	0.0000
SED	38	0.6500	0.9890	0.3490	14.1810	0.6340	0.0010	0.0000	0.3650	0.0000
SEA	31	0.7030	0.9880	0.2430	35.7980	0.7420	0.0110	0.0000	0.0000	0.2470
SHD	22	0.5780	0.9890	0.1830	89.3410	0.3070	0.0000	0.4490	0.2430	0.0000
SHA	2	0.5800	0.7950	0.5680	81.4850	0.3130	0.0000	0.4980	0.0000	0.1900
SDA	30	0.7170	0.9890	0.2320	40.3890	0.5640	0.0000	0.0000	0.2910	0.1450
EDA	11	0.4240	0.7380	0.7250	8.4650	0.0000	0.0200	0.0000	0.7640	0.2150
**EHA** ∗	**11**	**0.5770 **	**0.9800 **	**0.1990 **	**148.9890 **	**0.0000 **	**0.0630 **	**0.6910 **	**0.0000 **	**0.2460 **
HAD	2	0.5550	0.8020	0.5580	85.2150	0.0000	0.0000	0.6150	0.2080	0.1770
SEHD	23	0.5970	0.9890	0.1870	81.1730	0.2940	0.0230	0.4520	0.2310	0.0000
SEHA	23	0.5970	0.9800	0.1880	80.4080	0.3000	0.0420	0.4620	0.0000	0.1960
SEDA	31	0.7110	0.9890	0.2420	36.1870	0.5640	0.0050	0.0000	0.2840	0.1470
SHDA	5	0.5630	0.9290	0.3470	102.3970	0.2600	0.0000	0.3980	0.1920	0.1510
**EHDA** ∗	**12**	**0.6070 **	**0.9820 **	**0.1940 **	**143.5670 **	**0.0000 **	**0.0500 **	**0.5880 **	**0.2040 **	**0.1580 **
SEHDA	23	0.6120	0.9890	0.1880	80.3300	0.2690	0.0340	0.4020	0.1630	0.1330

OCN: Optimal number of components.

SEE: Standard error of estimate.

*F*: *F*-test value.

*Prediction model.

S: Steric.

H: Hydrophobic.

D: Hydrogen bond donor.

A: Hydrogen bone acceptor.

E: Electrostatic.

**Table 5:** Experimental and predicted pIC50 values of 45 DHFR inhibitors using the constructed CoMFA and CoMSIA models.

DHFR inhibitors no.	Experimental pIC50	CoMFA		CoMSIA_EHDA		CoMSIA_EHA	
		Predicted	Residual	Predicted	Residual	Predicted	Residual
1	4.710	4.652	0.0580	4.481	0.229	4.532	0.178
2	4.609	4.606	0.0031	4.635	−0.026	4.662	−0.053
3∗	4.231	4.576	−0.3454	4.333	−0.102	4.407	−0.176
4	4.662	5.027	−0.3655	4.698	−0.037	4.701	−0.039
5∗	4.524	4.571	−0.0467	4.807	−0.283	4.797	−0.273
6	4.893	4.476	0.4168	4.723	0.170	4.651	0.242
7∗	7.161	6.810	0.3512	7.287	−0.126	7.359	−0.198
8	6.810	6.529	0.2807	6.722	0.088	6.723	0.087
9∗	6.261	6.495	−0.2338	6.270	−0.009	6.240	0.021
10	6.873	6.648	0.2249	6.808	0.065	6.832	0.041
11	6.762	6.793	−0.0310	6.686	0.076	6.645	0.117
12	5.747	5.749	−0.0019	5.767	−0.020	5.705	0.042
13	5.273	5.346	−0.0727	5.245	0.028	5.279	−0.006
14	7.509	7.454	0.0546	7.494	0.015	7.522	−0.013
15	8.046	8.322	−0.2762	8.056	−0.010	8.052	−0.006
16	7.699	8.127	−0.4280	8.130	−0.431	8.110	−0.411
17	7.495	7.670	−0.1751	7.871	−0.376	7.820	−0.325
18	8.222	8.079	0.1428	8.130	0.092	8.105	0.117
19	7.569	7.561	0.0076	7.581	−0.012	7.609	−0.040
20	8.097	8.207	−0.1101	8.105	−0.008	8.240	−0.143
21	8.155	8.007	0.1479	8.242	−0.087	8.215	−0.060
22	8.699	8.127	0.5720	8.130	0.569	8.110	0.589
23∗	7.377	7.636	−0.2592	7.325	0.052	7.318	0.059
24	8.155	7.670	0.4849	7.871	0.284	7.820	0.335
25	6.807	7.113	−0.3061	6.902	−0.095	6.824	−0.017
26	7.959	7.987	−0.0284	7.887	0.072	7.975	−0.016
27	7.824	7.763	0.0609	7.981	−0.157	7.955	−0.131
28	7.854	7.839	0.0149	7.906	−0.052	7.850	0.004
29	7.824	7.843	−0.0191	7.824	0.000	7.827	−0.003
30	7.745	7.914	−0.1693	7.736	0.009	7.733	0.012
31	7.658	8.069	−0.4114	7.665	−0.007	7.654	0.004
32	8.222	8.005	0.2168	7.848	0.374	7.814	0.408
33∗	8.000	8.100	−0.1000	7.978	0.022	8.010	−0.010
34	7.886	7.455	0.4311	7.947	−0.061	7.811	0.075
35	8.222	7.981	0.2408	8.208	0.014	8.237	−0.015
36∗	8.000	8.173	−0.1730	8.130	−0.130	8.139	−0.139
37	8.155	8.180	−0.0251	8.170	−0.015	8.187	−0.032
38	8.097	8.122	−0.0251	8.097	0.000	8.097	0.000
39∗	8.000	7.990	0.0100	8.007	−0.007	8.054	−0.054
40	7.770	7.683	0.0866	7.832	−0.062	7.697	0.073
41	7.959	8.223	−0.2644	7.883	0.076	7.907	0.052
42	8.097	7.974	0.1229	8.040	0.057	8.150	−0.053
43	8.046	7.996	0.0498	8.052	−0.006	8.061	−0.015
44∗	7.387	7.542	−0.1548	7.567	−0.180	7.590	−0.203
45	7.495	7.449	0.0459	7.484	0.011	7.516	−0.021

*test set.

**Table 6:** Predicted bioactivity (pIC50) of MTX and TCM candidates using MLR, Bayesian, SVM, CoMFA and CoMSIA models.

Name	MLR	Bayesian	SVM	CoMFA	CoMSIA_EHDA∗	CoMSIA_EHA∗∗
Adenosine triphosphate	6.4559	5.8145	8.7175	7.9640	7.8600	7.8350
Methyl 6-O-digalloyl-beta-D-glucopyranoside (II)	27.5044	5.1810	8.0157	6.9800	6.6030	5.5170
Methyl 4,6-di-O-galloyl-beta-D-glucopyranoside	27.7317	5.4868	8.4131	7.5490	6.5980	5.9300
Methyl 6-O-digalloyl-beta-D-glucopyranoside	26.7188	5.2477	7.8936	6.8980	6.6620	5.6840
Manninotriose	29.1034	5.1934	5.9247	7.6470	6.2450	5.3700
Forsythiaside	29.9821	5.3595	8.5713	7.7140	8.0830	7.8950
Isoacteoside	27.6319	6.3265	8.1255	7.6550	7.7990	7.5430
Rehmannioside B	26.7291	4.3032	7.3293	6.9990	6.8000	5.8300
Rehmannioside A	30.3632	4.4182	9.3324	6.7480	5.8070	4.6750
Raffinose	32.8592	5.1647	8.4766	6.9350	5.9620	4.2830
Cistanoside C	26.1802	5.7174	8.2029	7.6060	8.0200	7.9640
Methyl 3,3,6-tri-O-galloyl-beta-D-glucopyranoside	30.7405	6.0369	8.3193	6.7670	6.3240	6.6300
Stachyose	40.5491	5.9779	8.5055	7.4300	5.6830	4.4510
Chlorogenic acid	17.3951	4.2335	7.8897	7.8080	7.9640	7.7680
Jionoside D	26.0421	5.5238	8.2089	7.5080	7.4900	7.2820
Isochlorogenic acid	16.1484	4.4196	7.4839	7.1990	6.3590	6.4480
Jionoside C	23.7203	5.6640	8.2741	7.7600	7.0800	6.9110
Rutin	30.3096	5.6910	8.2465	6.5720	8.0190	7.6830

The pIC50 experimental values of MTX was 8.5229.

*EHDA model of CoMSIA.

**EHA model of CoMSIA.
